# Angiotensin Receptor Autoantibodies in Dupuytren Disease: A Biomarker Study

**DOI:** 10.1177/22925503251344305

**Published:** 2025-06-05

**Authors:** Natasha D. Osborne, Julia M. Harrison, David Tang, Nadim G. Joukhadar, Michael Bezuhly

**Affiliations:** 1 Department of Microbiology and Immunology, 3688 Dalhousie University, Halifax, Nova Scotia, Canada; 2 Division of Plastic Surgery, 3688 Dalhousie University, Halifax, Nova Scotia, Canada; 3 Division of Plastic Surgery, 8166 University of British Columbia, Vancouver, British Columbia, Canada; 4 Division of Plastic Surgery, IWK Health Centre, Halifax, Nova Scotia, Canada

**Keywords:** angiotensin receptors, autoantibodies, biomarker, Dupuytren disease, auto-anticorps, biomarqueur, maladie de Dupuytren, récepteurs de l’angiotensine

## Abstract

**Background:** Dupuytren disease (DD) is a fibroproliferative disorder characterized by excess collagen deposition in the digitopalmar fascia resulting in disabling flexion contractures. The angiotensin II type 1 receptor (AT1R) pathway has previously been shown to be upregulated in a variety of other fibrotic disorders. We explored the potential association between DD and activating autoantibodies (AAb) against the profibrotic AT1R or counterregulatory antifibrotic angiotensin II type 2 receptor (AT2R). **Methods:** Patients with DD and controls were recruited from a single hand clinic. Demographic and clinical data and total flexion deformity angle of each digit were recorded. Serum levels of AT1R-AAb and AT2R-AAb were measured by enzyme-linked immunosorbent assay. **Results:** No differences were noted in serum AT1R-AAb levels between control and DD patients. In women with DD, circulating AT2R-AAb were significantly lower than in control women (7.61 ± 3.0 U/mL vs 13.5 ± 3.1 U/mL, respectively). AT2R-AAb observed values tended to be lower in women with higher Tubiana severity scores. In contrast, AT2R-AAb levels were not different between control or DD male subjects. **Conclusions:** These early findings suggest angiotensin II signaling differences may contribute to sex differences in DD and that an AT2R agonist may be particularly beneficial in treating women with DD.

**Level of Evidence:**

Diagnostic, Level II.

## Résumé

**Historique :** La maladie de Dupuytren (MD) est un trouble fibroprolifératif caractérisé par un dépôt excédentaire de collagène dans le fascia palmaire et digital, qui entraîne des contractures en flexion invalidantes. Il a déjà été démontré que la voie du récepteur de type 1 de l’angiotensine II (AT1R) est régulée à la hausse dans divers autres troubles fibreux. Les auteurs ont exploré l’association potentielle de la MD et des auto-anticorps d’activation (AAb) contre l’AT1R profibrotique ou le récepteur de type 2 de l’angiotensine II (AT2R). **Méthodologie :** Les auteurs ont recruté des patients atteints de la MD et des sujets témoins dans une seule clinique de la main. Ils ont enregistré les données démographiques et cliniques et l’angle de déformation en flexion totale de chaque doigt en flexion. Ils ont mesuré les taux de sérum d’AT1R-AAb et d’AT2R-AAb par dosage immuno-enzymatique. **Résultats :** Les auteurs n’ont remarqué aucune différence dans les taux d’AT1R-AAb sérique des sujets témoins et des patients atteints de la MD. Chez les femmes atteintes de cette maladie, l’AT2R-AAb en circulation était considérablement plus faible que chez les femmes faisant partie des sujets-témoins (7,61 ± 3,0 U/mL par rapport à 13,5 ± 3,1 U/mL). Les valeurs de l’AT2R-AAb observées avaient tendance à être plus faibles chez les femmes dont la classification de Tubania était plus élevée. En revanche, les taux d’AT2R-AAb n’étaient pas différents entre les sujets témoins et les sujets de sexe masculin atteints de la MD. **Conclusions :** D’après ces observations précoces, les différences de signalisation de l’angiotensine II peuvent contribuer aux différences de la MD en fonction des sexes et un agoniste de l’AT2R peut être particulièrement bénéfique pour traiter les femmes atteintes de la MD.

## Introduction

Dupuytren disease (DD) is a progressive fibroproliferative disorder affecting the digitopalmar fascia.^1^ The incidence of DD increases with age and is more common in males, with men 2 to 4 times more likely to be affected than women.^2,3^ As DD is a fibrotic condition, one possible approach for prevention or treatment is to modulate profibrotic cellular signaling pathways. One such signaling pathway is the renin–angiotensin system. Outside of its canonical function as a circulating hormone regulating systemic blood pressure and salt/water homeostasis, angiotensin II also induces local fibrosis through the angiotensin II type 1 receptor (AT1R).^4,5^ AT1R activation induces an intracellular signaling cascade resulting in fibroblast proliferation and differentiation, leading to extracellular matrix protein synthesis and deposition, as well as inflammation.^4^ Importantly, fibrotic diseases such as systemic sclerosis have been associated with increased circulating autoantibodies (AAbs), which activate AT1R,^6^ and targeting AT1R with the angiotensin receptor blocker (ARB) losartan has shown to reduce fibrosis in pulmonary,^7^ and hepatic fibrosis.^8^ Furthermore, preformed AT1R-AAbs in serum of patients undergoing renal,^9^ hepatic,^10^ or cardiac^11^ transplants have been found to be predictive of an increased risk of chronic allograft rejection. As well, patients who develop de novo AT1R-AAbs after organ transplant have an increased risk of complications, including fibrosis in the donor tissue and graft failure.^11,12^ The sustained AAb activation of AT1R likely induces chronic inflammation and fibrosis in the transplant tissue to contribute to graft loss.^12,13^ Importantly, treatment with losartan has shown some efficacy in counteracting AT1R-AAb elevations and mitigating graft complications and loss.^13^ However, given that AT1Rs are widely expressed throughout the body, medications targeting this receptor pose the risk of off-target effects, including hypotension.^4,14^

In contrast, activation of angiotensin type 2 receptors (AT2Rs) counterregulates AT1R signaling by reducing TGF-β1 expression, inflammation and extracellular matrix accumulation.^4^ Importantly, unlike AT1Rs, these receptors are not constitutively expressed on fibroblasts, and are upregulated in areas of tissue injury and remodeling, making them a more attractive therapeutic target. Much less is known regarding the role of AT2R signaling and its associated AAbs in fibrotic conditions. AT2R is upregulated in tissues taken from patients with idiopathic pulmonary fibrosis,^15^ likely to counter ongoing profibrotic signaling. High levels of AT2R expression have been observed in DD patient tissues, indicating that AT2R may be a particularly relevant target in this disease.^16–18^ We have shown that selective AT2R agonist compound 21 (C21) reduced myofibroblast activation in a mouse xenograft model of DD.^18^

The role of circulating agonist AAbs against AT1R and AT2R has not been examined in the context of DD. Given the important role of fibrosis signaling pathways in DD and the evidence for AT2R expression in patient tissue, we postulated that circulating angiotensin II receptor AAbs may be altered in individuals with DD. We therefore quantified AT1R- and AT2R-AAb levels from sera collected from DD patients to determine whether an association existed between these AAb and DD that was amenable to future pharmacological targeting.

## Methods

### Patient Recruitment

The prospective cohort study received full approval and was conducted in accordance with its policies. Patients presenting to the hand surgery clinic at a single tertiary care center between January 2020 and December 2022 with DD were approached by a research coordinator for participation in the study. The control group consisted of patients assessed for basal thumb osteoarthritis in the same clinic with no history of DD. Informed written consent was obtained. Inclusion criteria was diagnosis of DD or basal thumb osteoarthritis (control) and age 18 years or older. Exclusion criteria included concomitant fibrotic disease and age younger than 18 years.

### Data Collection

Patient demographic and clinical information collected included the following: age, sex, gender, medical history (number of years with disease, previous treatments, comorbidities, and family history), medications, and use of cigarettes, alcohol, and/or recreational drugs.

For patients with DD, the number of digits and/or palmar rays affected by the disease was noted, and the total flexion deformity (TFD) angle of each digit was measured by goniometer at the metacarpalphalangeal (MCPJ), proximal (PIPJ), and distal interphalangeal (DIPJ) joints. A modified Tubiana severity score was then calculated for each patient.^19^ Each digit was given a score ranging from 0 to 4, depending on the severity of disease. Palmar nodules without presence of contracture were scored as 0.5. For digits with contracture, TFD was converted to a score of 1 (TFD between 0° and 44°), 2 (TFD between 45° and 89°), 3 (TFD between 90° and 134°), or 4 (TFD greater than 135°).^20^ Digits amputated or that underwent previous surgery for DD were given the maximum score of 4. The 10 scores generated from the individual digits were then summed to generate a total score for each patient. Blood samples (30 mL) were collected in clinic by a nurse, and the serum separated by centrifugation and immediately stored at −80 °C prior to being transported for further analysis in the senior author's laboratory. All blood samples were provided with a unique identifier number, and their group (DD vs control) masked.

### Enzyme-Linked Immunosorbent Assay

Serum AT1R- and AT2R- AAb levels were quantified using enzyme-linked immunosorbent assay (ELISA) kits for human angiotensin II receptor 1 agonist autoantibody (Novus Biologicals, LLC, Centennial, CO) and human angiotensin II receptor 2 agonist autoantibody (Cusabio Technology LLC, Houston, TX) as per the manufacturer's instructions. Briefly, serum samples were diluted 1:4 in the diluent provided. Standards were prepared by 2-fold serial dilution from the reconstituted stock reference standard (20 ng/mL or 20 U/mL for AT1R-AAb and AT2R-AAb kits, respectively), with the diluent serving as the 0 standard. Equal volumes of samples or standards were then added to the supplied precoated ELISA plates. Wells were washed and unbound biotin-conjugated antihuman AT1R-AAb or AT2R-AAb antigen were added and allowed to incubate for 1 h at 37 °C. Following a second round of washes, HRP-avidin conjugate was added, incubated for 30 to 60 min, and wells washed again. Carbamide peroxide (AT1R-AAb ELISA) or tetramethylbenzidine (AT2R-AAb ELISA) substrate reagent was added to each well and incubated for 30 min. Stop solution (sulfuric acid) was then added, and plates immediately read using a BioTek Synergy HTX multimode microplate reader (BioTek Instruments, Winooski, VT). For AT1R-AAb, the plate was read at 450 nm. For AT2R-AAb, the plate was first read at 570 nm to correct for optical imperfections in the plate, followed by 450 nm, as per manufacturer's instructions. The 570 nm and blank sample absorbances were subtracted from the 450 nm measurement. The average of the duplicates for each sample/standard was then calculated. Sample AT1R-AAb and AT2R-AAb concentrations (ng/mL or U/mL, respectively) were interpolated from the standard curve and multiplied by 4 to account for the 1:4 sample dilution.

### Statistical Analysis

A sample size was calculated based on a previous report examining AT2R-AAb titres.^21^ Assuming a 20% difference in AT2R-AAb titres between DD and control groups with mean titre level 17 U/mL, standard deviation 2 U/mL, alpha 0.05, and power 0.80, 16 subjects would be required per group. To account for attrition, target recruitment was set at 20 subjects per group. All data were first assessed for normality by Shapiro–Wilk and Kolmogorov–Smirnov tests, and homogeneity of variance (F-test and Bartlett's tests). Patient demographics data were all analyzed using Fisher's exact tests, except for patient age, which was analyzed using unpaired 2-tailed *t*-test. DD characteristics were analyzed by unpaired *t*-test with Welch's correction (due to heterogeneity of variances for Tubiana severity scores), nonparametric Mann–Whitney U tests (number of surgeries and number of digits affected), or nonparametric Fisher's exact test (bilateral disease). The initial comparison of AAb levels between all control and DD patients was performed using unpaired 2-tailed t-test (AT1R) or Mann Whitney U test (AT2R). The concentrations of AT1R- and AT2R-AAbs were analyzed by 2-way ANOVAs between DD and controls and between sexes. Specific group comparisons were conducted with a Sidak correction to maintain the family-wise error rate at 0.05. Pearson's *r* (parametric) or Spearman's *r* (nonparametric) correlations were run as appropriate comparing AT1R-AAb and AT2R-AAb levels to Tubiana severity score. Statistical analyses were performed using GraphPad Prism 8.4.2 for all analyses (Dotmatics, IBM, Boston, MA).

## Results

Of the 41 patients recruited, a total of 39 patients were included in the analysis as one DD male and one control male completed the questionnaire but did not have their serum collected. As shown in Table 1, our study population consisted of 14 male and 8 female DD patients, and 13 male and 4 female control patients. All patients identified as cis-gendered. The average age and sex distribution of control and DD groups were comparable, and there were no differences in the proportions of diabetes, hypertension, or dyslipidemia comorbidities between control and DD groups. A higher percentage of control patients were on lipid-lowering medication (*p* = .011) compared to DD patients. While there was a trend for female DD patients to have undergone a higher number of previous DD-specific procedures (needle aponeurotomy, collagenase injection or fasciectomy; *p* *=* .074), no differences were identified between male and female subjects in terms of Tubiana score, number of affected digits, or incidence of bilateral disease.

**Table 1.** Patient Demographics and Clinical Characteristics.

Sample characteristics	All subjects (*n* = 39)	Controls (*n* = 17)	DD (*n* = 22)	*p*-value
Age, mean (SD)	65.6 (7.3)	66.4 (6.9)	65.0 (7.7)	.57
Sex, *n* (%)				
Males	27 (69.2%)	13 (76.5%)	14 (63.6%)	.49
Females	12 (30.8%)	4 (23.5%)	8 (36.4%)	
Medical history, *n* (%)				
Comorbidities				
Diabetes	7 (18.0%)	5 (29.4%)	2 (9.1%)	.21
Hypertension	23 (59.0%)	11 (64.7%)	12 (54.6%)	.74
Dyslipidemia	20 (51.3%)	11 (64.7%)	9 (40.9%)	.20
Pharmacological agent use, *n* (%)				
Diabetes medication	7 (18.0%)	5 (29.4%)	2 (9.1%)	.21
Hypertension medication				
Angiotensin-converting enzyme inhibitor	9 (23.1%)	5 (29.4%)	4 (18.2%)	.47
AT1R antagonist	8 (20.5%)	4 (23.5%)	4 (18.2%)	.71
Other	18 (46.2%)	8 (47.1%)	10 (45.5%)	>.99
Lipid lowering medication	16 (41.0%)	11 (64.7%)	5 (22.7%)	**.** 011*
Antiinflammatory	8 (20.5%)	6 (35.3%)	2 (9.1%)	.059
Other	27 (93.1%)	14 (82.4%)	13 (59.1%)	.17
No prescription medications	9 (23.1%)	1 (5.9%)	8 (36.4%)	.052
Current cigarette smoker, *n* (%)	12 (30.8%)	8 (47.1%)	4 (18.2%)	.082
Current alcohol user, *n* (%)	29 (74.4%)	10 (58.8%)	18 (81.8%)	.16
Current use of cannabis or recreational drugs, *n* (%)	5 (12.8%)	2 (11.8%)	3 (13.6%)	>.99

**Figure 1.**
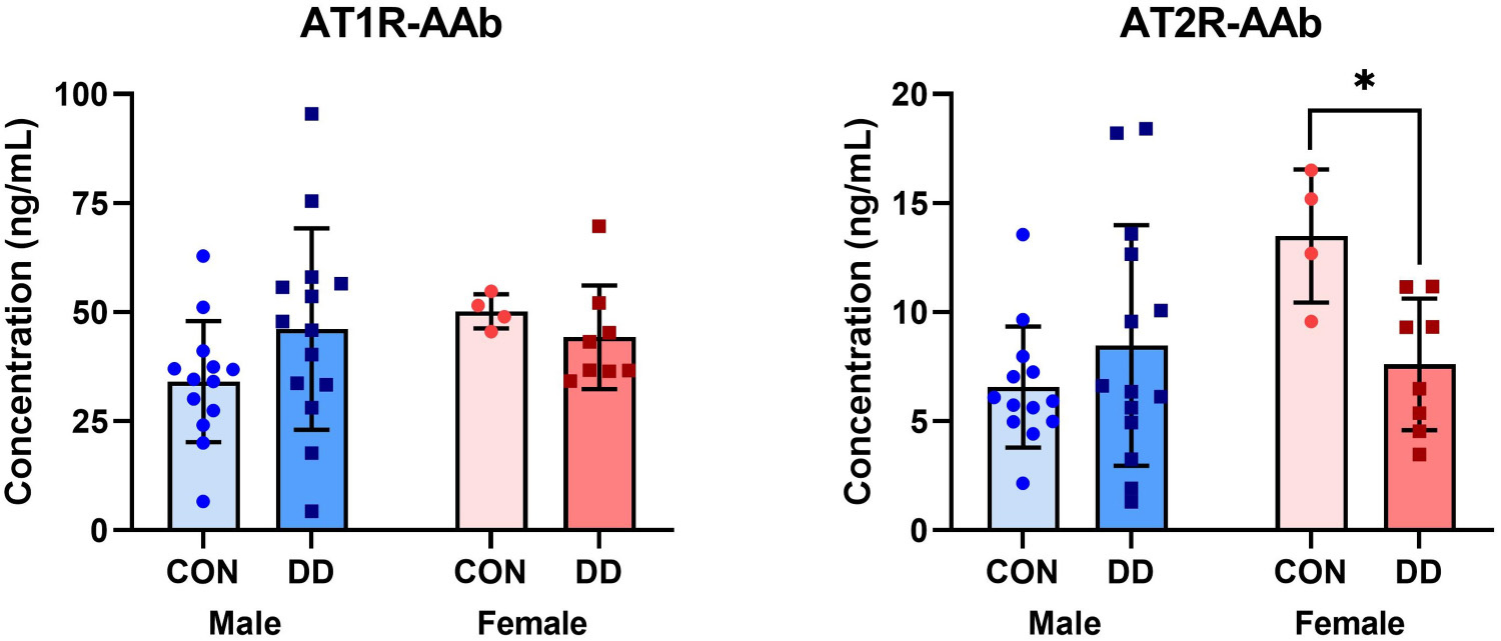
Bar charts (Mean ± SD) depicting concentrations of AT1R- and AT2R-AAbs measured from sera samples collected from male and female control or DD patients. AT1R- and AT2R-AAb levels were measured in duplicate via separate ELISAs according to the manufacturer's instructions. Female DD patients had significantly lower circulating levels of AT2R-AAb relative to female controls, *p = .047. *AT1R-AAb,* Angiotensin II Type 1 receptor autoantibodies; *AT2R-AAb,* Angiotensin II Type 2 receptor autoantibodies; *CON,* control; *DD*, Dupuytren disease; ELISA, enzyme-linked immunosorbent assay.

AT1R-AAb titers between all DD patients (45.48 ± 19.43) and controls (37.90 ± 14.03) did not differ. Similarly, no difference was observed in AT2R-AAb titers between all DD patients and controls (6.56 [IQR 4.82, 11.16] and 7.05 [IQR 5.30, 11.17], respectively). Given DD is known to affect men more frequently than women,^2,3^ we next compared AAb titers, stratifying by sex (Figure 1). Although serum AT1R-AAb levels were comparable between control and DD patients in both males and females, women with DD had significantly lower circulating AT2R-AAb levels (mean difference −5.89, 95% CI [−10.02, −1.75]) compared to their control counterparts (*p* *=* .047).

**Figure 2.**
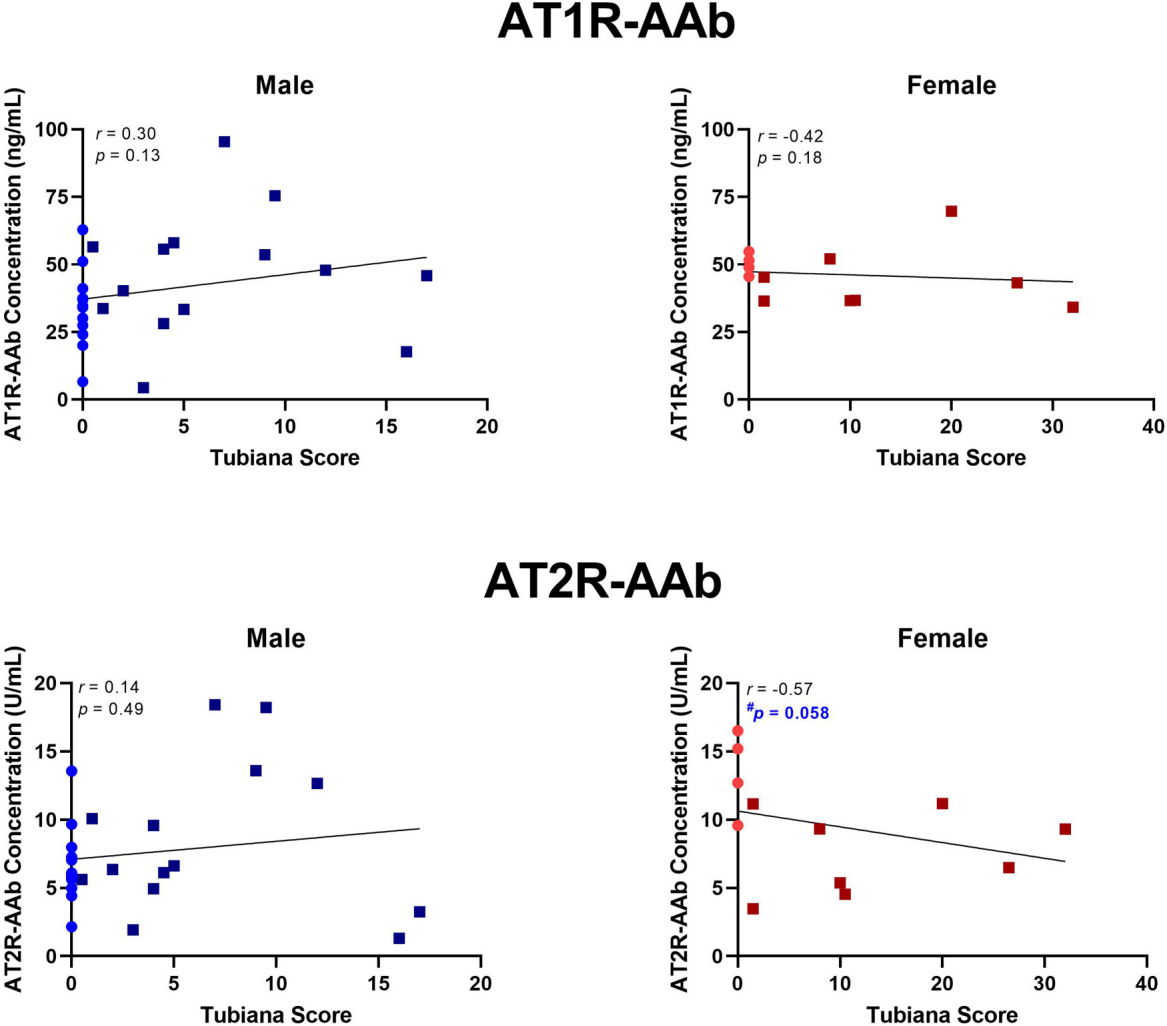
AT1R-AAb and AT2R-AAb concentrations in relation to Tubiana severity scores. Spearman's *r* was computed for all 4 correlations. AT1R-AAb levels did not correlate with Tubiana scores, in either males or females. In females, there was a trend for a negative correlation between Tubiana severity score and AT2R-AAb concentration, *r* = 0.57*, p* = .058. *AT1R-AAb,* Angiotensin II Type 1 receptor autoantibodies; *AT2R-AAb,* Angiotensin II Type 2 receptor autoantibodies.

No statistically significant correlations were identified between Tubiana severity score and AT1R-AAb titres in male or female patients, nor AT2-AAb levels in males. Though not statistically significant, AT2R-AAb concentrations tended to be lower in female patients with high Tubiana severity scores, *r* *=* −0.57, *p* *=* .058 (Figure 2).

## Discussion

Given evidence of AT1R-AAbs upregulation in other fibrotic conditions^6–8^ and AT2R receptor expression changes in DD,^16–18^ we investigated whether AAbs against AT1R and AT2R were altered in patients with DD. Although the role of AT2R in fibrotic diseases is less well understood than AT1R, its activation by angiotensin II is known to induce antifibrotic signaling cascades, whereby inflammation and the production of collagen and other extracellular matrix factors are reduced.^4,21^ Thus, we speculate that low circulating levels of AT2R-AAb and the subsequent decreased stimulation of this “protective” arm of the renin–angiotensin system may contribute to the accumulation of fibrotic tissue in DD. This points to a novel avenue to be explored for improved DD treatment, namely drugs which boost antifibrotic AT2R signaling. Indeed, we have previously shown that treatment with the selective AT2R agonist compound 21 reduced fibrosis in a murine xenograft model of DD.^18^

Considering DD affects men far more frequently than women,^2,3^ the observation that AT2R-AAb titers were lower in women with DD is interesting. To date, research examining the significant sex bias of DD has been limited. Some have speculated that the observed 2 to 4:1 ratio of men to women with DD may be due to reporting bias, as men are more likely to present with more aggressive and recurrent forms of the disease requiring surgery.^3,22,23^ There is evidence that premenopausal women are less likely to develop severe fibrotic diseases^24^ due to the protective effects of estrogen, which has been shown to reduce profibrotic AT1R expression and increase antifibrotic AT2R density.^25^ We did not observe a baseline difference in Tubiana severity scores between men and women in our patient cohort; however, when disease severity was correlated against AT2R-AAb concentration, we found that females with a high Tubiana score tended to have lower serum AT2R-AAb levels (Figure 2). These findings lead us to speculate that downregulation of the “protective” antifibrotic arm of the renin–angiotensin system may contribute to DD in women. Additional research is required into the role which biologic sex plays in DD development, recurrence, and potential responses to treatment.

The principal limitation of our study were limited subjects for the gender subanalyses performed as our *a priori* sample size calculation was based on all DD and control subjects. By chance alone, one would expect 1 out of 20 analyses to be significant, but of the 10 performed (6 in Figure 1; 4 in Figure 2) one reached significance with appropriate correction for multiple comparisons (circulating AT2R-AAbs in women with DD vs controls), and one almost met significance (correlations between AT2R-AAbs and age and Tubiana severity scores). While by no means definitive, this points to an association between AT2-AAbs and DD in women that warrants further investigation.

Future directions include expansion of the current exploratory study to include prospective sample collections from multiple centers to allow for more robust assessment for a potential dose effect of AAb titers in both sexes. Our observation that AT1R-AAbs did not differ between DD and control populations suggests that clinically available AT1R blockers such as losartan are unlikely to be of benefit in DD patients. Instead, our work provides proof-of-principle for clinical studies examining the use of a pharmacologic AT2R agonist such as compound 21 (currently only approved for clinical use in Europe) in reducing DD disease burden. This novel treatment avenue may be particularly effective in women with DD, as females may be more likely to develop posttreatment complications and exhibit poorer outcomes compared to men.^20^
